# Who is eligible for randomized trials? A comparison between the exclusion criteria defined by the ISCHEMIA trial and 3102 real-world patients with stable coronary artery disease undergoing stent implantation in a single cardiology center

**DOI:** 10.1186/s13063-015-0934-4

**Published:** 2015-09-15

**Authors:** Jarosław Wasilewski, Lech Poloński, Andrzej Lekston, Tadeusz Osadnik, Rafał Reguła, Kamil Bujak, Anna Kurek

**Affiliations:** Medical University of Silesia, School of Medicine with the Division of Dentistry, 3rd Department of Cardiology, Silesian Center for Heart Diseases, Marii Skłodowskiej-Curie Street 9, 41-800 Zabrze, Poland

**Keywords:** ISCHEMIA trial, Revascularization versus optimal medical treatment, Stable coronary artery disease

## Abstract

**Background:**

Randomized controlled trials are the gold standard for evaluating therapy; however, controversy exists regarding the applicability of such results to daily practice, as patients are often pre-selected and may not reflect real-world clinical settings. We studied the eligibility criteria for 3102 “real-life” patients with stable coronary artery disease (SCAD) according to the ISCHEMIA (International Study of Comparative Health Effectiveness with Medical and Invasive Approaches) trial exclusion criteria. The aim of our analysis was to estimate the percentage of real-life patients who would have met the exclusion criteria for the ISCHEMIA trial.

**Methods:**

We analyzed 3102 patients with SCAD referred to the Silesian Center for Heart Disease who underwent both coronary angiography and stent implantation between January 2006 and December 2011. The patients were divided into two groups. Group A was composed of patients with SCAD who would have been excluded from the ongoing ISCHEMIA trial, whereas group B represented the remaining patients.

**Results:**

A total of 1900 (61.3 %) patients met at least one of the exclusion criteria. The most frequent exclusion criterion noted was revascularization within the previous 12 months (938 patients; 49.4 %), followed by unacceptable level of angina symptoms (532 patients; 28 %), low ejection fraction (467 patients; 24.6 %), and acute coronary syndrome within the previous 2 months (456 patients; 24 %). Patients from our cohort who would have been excluded from the ISCHEMIA trial were older, had more comorbidities, and experienced worse long-term outcomes.

**Conclusions:**

The ISCHEMIA trial exclusion criteria ruled out the majority of the patients with SCAD undergoing percutaneous coronary intervention in “real life”. Our cohort of patients who would have been excluded from the ISCHEMIA trial had more comorbidities and experienced significantly worse long-term outcomes than patients who did not meet the ISCHEMIA trial exclusion criteria.

**Trial registration:**

ClinicalTrials.gov NCT01471522.

**Electronic supplementary material:**

The online version of this article (doi:10.1186/s13063-015-0934-4) contains supplementary material, which is available to authorized users.

## Background

Stable coronary artery disease (SCAD) remains one of the most common indications for referral to a cardiac catheterization laboratory [1]. Primary treatment goals in patients with SCAD include the prevention of acute coronary syndrome and the relief of ischemia. Percutaneous coronary intervention and coronary artery bypass grafting are established methods of improving cardiac symptoms [2, 3].

The benefits of revascularization remain unclear. The COURAGE (Clinical Outcomes Utilizing Revascularization and Aggressive Drug Evaluation) trial demonstrated no difference in mortality between patients with SCAD who were treated invasively and those who treated using optimal medical therapy [4]. Although the COURAGE trial was composed of a wider spectrum of patients than previous studies [5–11], there were concerns regarding its design, related to potential selection biases [12]. A meta-analysis by Boden *et al.* [4] demonstrated that, in patients with SCAD, percutaneous coronary intervention did not offer any benefit in terms of mortality, incidence of myocardial infarction or need for subsequent revascularization over optimal medical therapy; however, a more recent meta-analysis by Windecker *et al.* [13] provided evidence regarding improved survival with the use of new-generation drug-eluting stents as opposed to balloon angioplasty, bare metal stents or early-generation drug-eluting stents.

Numerous trials have compared optimal medical therapy with revascularization for periods of up to 30 days [14–17], but all of them included cohorts selected via randomization. Therefore, the results of those studies may not be representative for the entire population of patients undergoing percutaneous coronary intervention in real life, particularly among subgroups of patients with a high baseline cardiovascular risk who are excluded from most randomized trials [17]. In a study that included low risk patients with SCAD, the use of an invasive strategy worsened the prognosis of myocardial infarction, stroke and cardiovascular death, as did the use of repetitive revascularization [18] and other techniques, suggesting modest benefits [19–21]. Therefore, selection bias and risk burden are crucial in establishing the suitability of invasive revascularization in a broad spectrum of patients with SCAD.

The purpose of the ongoing ISCHEMIA (International Study of Comparative Health Effectiveness with Medical and Invasive Approaches) trial is to determine the best management strategy for high-risk patients with stable ischemic heart disease and proven ischemia, using different diagnostic modalities. The primary aim of the ISCHEMIA trial is to test the hypothesis that the use of an invasive strategy, followed by revascularization plus optimal medical therapy, in patients with either moderate or severe ischemia inducible on stress imaging, is superior to a conservative strategy (optimal medical therapy only) [22].

In this analysis, we studied the eligibility criteria of 3102 consecutive patients with SCAD who underwent stent implantation, according to the exclusion criteria of the ISCHEMIA trial, to determine what percentage of real-world patients would be excluded from the ISCHEMIA trial. In addition, we characterized both the risk profiles and the long-term outcomes of patients who did not fulfill the exclusion criteria of the ISCHEMIA trial.

## Methods

We analyzed a cohort of 3502 patients with SCAD who were referred to the Silesian Center for Heart Disease (Zabrze, Poland) and underwent both coronary angiography and stent implantation between January 2006 and December 2011.

We screened all patients who underwent coronary angiography but were discharged with diagnosis other than SCAD (ICD10 I25.0 or I25.2) [23]. The screening was performed to identify patients admitted because of angina symptoms but discharged with another diagnosis (for example, cardiogenic shock) owing to in-hospital complications. Data regarding patients’ clinical and demographic characteristics, as well as their symptoms on admission, were taken from an electronic database containing data from structured medical charts. This database has been used to store information regarding patients’ medical histories at our institution since 2006. Patients’ echocardiography, angiography and laboratory test results were collected from the medical history database. All patients admitted to our center signed consent forms for data collection and processing and provided phone contact as part of our admission procedure. Patients who did not consent to phone contact were excluded from this analysis. All patients fulfilled the ISCHEMIA inclusion age criterion. The youngest analyzed patient was 29 years old. Out of a group of 3502 patients, 400 patients were excluded from further analysis due to incomplete data. A further study was conducted on a group of 3102 patients with complete clinical data. The study was approved by the ethics committee at the regional medical chamber (Ethics Committee of Silesian Medical Chamber, Katowice; Resolution number 34/2011 from 21 November 2011).

### Follow-up data

Information regarding survival was based on patients’ Polish National Health Fund insurance status, which may be verified electronically, as this national health insurance is mandatory for all Polish citizens; patients who were insured were considered to be alive. We attempted to contact the relatives of any uninsured patients, as well as the relevant local registry office, to obtain patients’ dates of death. Complete follow-up data were available for 3086 (99.5 %) patients. The median follow-up duration was 3.5 years. During the observation period, 366 deaths were reported.

### ISCHEMIA trial exclusion criteria

**Fig. 1:**
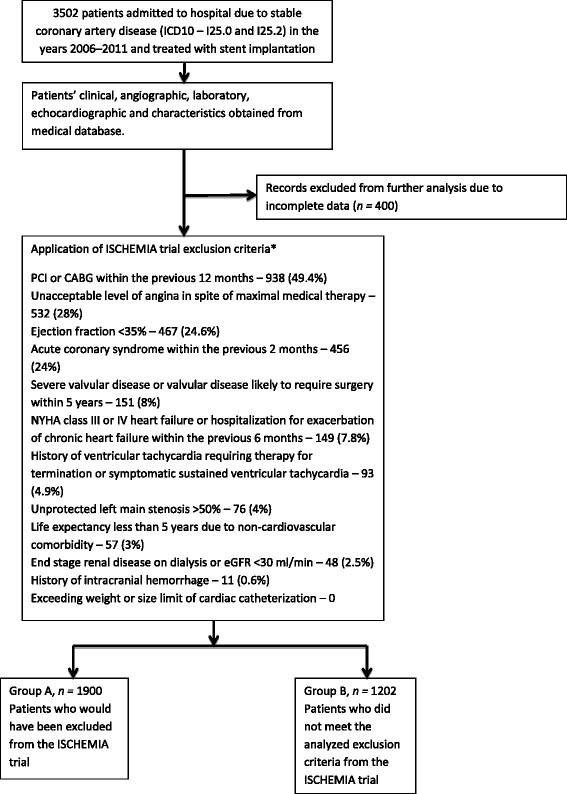
Scheme of the study. Owing to retrospective type of analysis or insufficient data, the following criteria were not applicable: finding of non-obstructive coronary artery disease, unsuitable coronary anatomy, pregnancy, patients with an estimated glomerular filtration rate of 30–59 who were likely to have significant unprotected left main stenosis, inability to comply with the ISCHEMIA protocol, very dissatisfied with medical treatment, ischemic stroke within 6-months, history of non-compliance

We analyzed the exclusion criteria using an ISCHEMIA study protocol available online. The exclusion criteria that we applied to the cohort are given in Fig. 1. Regarding the ISCHEMIA trial protocol, we did not note any restrictions pertaining to patients who underwent a previous valve replacement procedure. Therefore, we considered an implanted heart valve to be an exclusion criterion. The patients were divided into two groups. Group A was composed of patients with SCAD who would have been excluded from the ongoing ISCHEMIA trial, whereas group B represented the remaining patients who did not meet the ISCHEMIA exclusion criteria.

### Statistical analysis

Continuous variables are presented as medians and interquartile ranges. Categorical variables are presented as percentages. Continuous variables were compared using either the *t* test or the Mann–Whitney U test, where appropriate. Long-term prognoses were analyzed in both groups using the Kaplan–Meier method, with log-rank testing. All reported values of *P* are two-sided. The analyses were performed using Statistica, version 7.1 (StatSoft, Inc., Tulsa, OK, USA), and Number Crunching Statistical Systems, version 8 (NCSS; Kaysville, UT, USA).

## Results

**Fig. 2:**
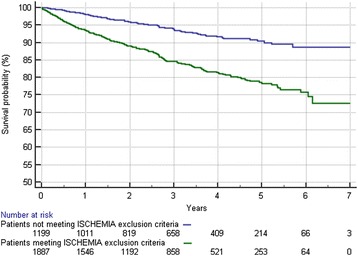
Kaplan–Meier survival plots of patients meeting the exclusion criteria for the ISCHEMIA trial (group A) and patients who would have been eligible for the ISCHEMIA trial (group B)

A total of 1900 (61.3 %) patients met at least one of the exclusion criteria pertaining to the ISCHEMIA trial. The most frequent exclusion criterion noted was revascularization within the previous 12 months (938 patients; 49.4 %), followed by unacceptable level of angina symptoms (532 patients; 28 %), low ejection fraction (467 patients; 24.6 %) and acute coronary syndrome within the previous 2 months (456 patients; 24 %) (Fig. 1). A total of 802 (44.2 %) patients fulfilled more than one exclusion criterion. The basic clinical characteristics of our cohort and the patients who met the exclusion criteria (group A), as well as those of the remaining patients (group B), are listed in Table 1. The patients who would have been excluded from the ISCHEMIA study (group A) were older and had more comorbidities, including myocardial infarction, previous revascularization, diabetes, hypertension, heart failure, and lower ejection fraction. These patients exhibited lower hemoglobin levels (median 8.5 mmol versus 8.8 mmol, *P* < 0.001) and hematocrits (median 41 % versus 42 %, *p* < 0.001), as well as lower estimated glomerular filtration rates (median 84.0 ml/min/ 1.73 m^2^) versus 85.2 ml/min/ 1.73 m^2^), *P* < 0.001) and higher creatinine levels (median 80.1 mmol/l vs. 77.7 mmol/l, *P* < 0.001) (Table 2). Group A also exhibited a higher incidence of multivessel disease (median 22.1 % versus 18.1 %, *P* = 0.01) (Table 3). Additionally, the patients who met at least one of the exclusion criteria pertaining to the ISCHEMIA trial had worse long-term prognosis. During the follow-up period, 79 (6.6 %) patients in group B and 287 (15.1 %) patients in group A, respectively, died. The mortality for the whole analyzed group was 11.8 % (366 patients). Figure 2 shows the Kaplan–Meier survival curves for cohorts who met the exclusion criteria for the ISCHEMIA trial (group A), as well as the data pertaining to the patients who would have been eligible for the ISCHEMIA trial (group B). Multivariate analysis of independent predictors of mortality in the analyzed group was presented in additiona material (Additional file 1).

**Table 1:** Baseline clinical characteristics, expressed as percentages or as medians and interquartile ranges

Variable		All patients	Group A	Group B	*P*
			(Patients who met ISCHEMIA exclusion criteria)	(Patients who did not meet ISCHEMIA exclusion criteria)	(A vs. B)
		*n* = 3102	*n* = 1900	*n* = 1202	
Median age, years		64 (57–71)	64 (57–72)	64 (57–70)	0.11
Men		2187 (70.5)	1354 (71.3)	833 (69.3)	0.26
Previous myocardial infarction		1786 (57.6)	1335 (70.3)	451 (37.5)	<0.001
Previous percutaneous coronary intervention		1451 (46.9)	1166 (61.4)	285 (23.7)	<0.001
Previous coronary artery bypass graft		407 (13.1)	253 (13.3)	154 (12.8)	0.7
Diabetes		1118 (36.1)	785 (41.3)	333 (27.8)	<0.001
Diabetes treatment	Diet only	238 (7.7)	1176 (9.3)	62 (5.2)	<0.001
	Insulin	433 (14.0)	284 (14.9)	149 (12.4)	
	Oral drugs	447 (14.4)	325 (17.1)	670 (55.7)	
Hypertension		2217 (71.5)	1301 (68.5)	916 (76.2)	<0.001
Hypercholesterolemia		1752 (56.5)	1082 (57)	670 (55.7)	0.53
Current smoker		1140 (36.9)	719 (37.8)	421 (35)	0.09
Past smoker		331 (10.7)	187 (9.8)	144 (12)	0.09
Obesity		1035 (33.4)	626 (33)	409 (34)	0.56
Family history of premature myocardial infarction (<55 years)		256 (9.3)	155 (8.3)	131 (10.9)	0.01
Systolic blood pressure (mmHg)		130 (120–140)	130 (120–140)	130 (120–140)	<0.001
Diastolic blood pressure (mmHg)		80 (70–85)	80 (70–85)	80 (70–85)	0.03
Heart rate (min^−1^)		70 (63–76)	70 (63–77)	70 (64–76)	0.32
Heart failure		568 (18.3)	496 (26.1)	72 (6)	<0.001
Ejection fraction (%)		48 (40–55)	45 (35–50)	50 (46–55)	<0.001
Mitral valve regurgitation, severe		75 (2.5)	75 (4.1)	0	<0.001
Mitral valve stenosis, severe		4 (0.1)	4 (0.2)	0	
Aortic stenosis, severe		24 (0.8)	24 (1.3)	0	
Aortic regurgitation, severe		7 (0.2)	7 (0.4)	0	<0.001
Aortic valve insufficiency, combined		5 (0.2)	5 (0.3)	0	
Bicuspid aortic valve		6 (0.2)	6 (0.3)	0	
Tricuspid regurgitation, severe		26 (0.9)	26 (1.4)	0	<0.001

**Table 2:** Laboratory data, expressed as medians and interquartile ranges

Variable	All patients	Group A	Group B	*P*
		(Patients who met ISCHEMIA exclusion criteria)	(Patients who did not meet ISCHEMIA exclusion criteria)	(A vs. B)
	*n* = 3102	*n* = 1900	*n* = 1202	
Red blood cell count (10^6^/µL)	4.5 (4.2–4.8)	4.4 (4.1–4.8)	4.6 (4.3–4.9)	<0.001
Hemoglobin (mmol/l)	8.6 (8–9.2)	8.5 (7.8–9)	8.8 (8.2–9.4)	<0.001
Hematocrit (%)	41 (38–45)	41 (38–45)	42 (39–45)	<0.001
White blood cells (10^3^/mm^3^)	7.2 (6.1–8.4)	7.1 (6–8.4)	7.2 (6.1–8.5)	0.49
Platelets (10^3^/mm^3^)	200 (163–240)	199 (163–241)	200 (164–235)	0.68
Creatinine (mmol/l)	79.2 (67.2–93.5)	80.1 (68.5–96)	77.7 (66–90)	<0.001
Estimated glomerular filtration rate (ml/min 1.73 m^2^))	83.3 (67.8–99.7)	82 (62.6–99.3)	85.2 (68.3–100.1)	<0.001

**Table 3:** Angiographic characteristics and procedural complications, expressed as percentages or as median and interquartile ranges

Variable		All patients	Group A	Group B	*P*
			(Patients who met ISCHEMIA exclusion criteria)	(Patients who did not meet ISCHEMIA exclusion criteria)	(A vs. B)
		*n* = 3102	*n* = 1900	*n* = 1202	
Angiographic characteristics					
Multivessel disease		636 (20.5)	419 (22.1)	217 (18.1)	0.01
Significant stenosis; left main coronary artery		157 (5.1)	129 (6.8)	28 (2.3)	<0.001
Significant stenosis – left anterior descending or diagonal		1781 (57.4)	1092 (57.5)	689 (57.3)	0.94
Significant stenosis – circumflex or obtuse marginal		1620 (52.2)	579 (48.2)	1041 (54.8)	<0.001
Significant stenosis – right coronary artery		1746 (56.3)	1045 (55)	701 (58.3)	0.07
Number of vessels, percutaneous coronary intervention		1 (1–1)	1 (1–1)	1 (1–1)	0.005
Number of stents		1 (1–2)	1 (1–2)	1 (1–2)	0.005
Implanted stent	bare metal stent	1785 (57.5)	1087 (57.2)	698 (58.1)	
	drug-eluting stent	1211 (39)	743 (39.1)	468 (38.9)	0.57
	bare metal stent and drug-eluting stent	106 (3.5)	70 (3.7)	36 (3)	
Complications					
Myocardial infarction		40 (1.3)	27 (1.4)	13 (1.1)	0.51
Stroke or transient ischemia attack		10 (0.3)	9 (0.5)	1 (0.1)	0.1
Bleeding		52 (1.7)	29 (1.5)	23 (1.9)	0.47
Blood transfusion		30 (1.0)	22 (1.2)	8 (0.7)	0.19
Dissection		159 (5.1)	108 (5.7)	51 (4.2)	0.08
Repeat of percutaneous transluminal coronary angioplasty		9 (0.3)	5 (0.3)	4 (0.3)	0.74
Urgent coronary artery bypass graft		2 (0.1)	1 (0.1)	1 (0.1)	1.0
Sudden cardiac arrest		30 (1)	16 (0.8)	14 (1.2)	0.45
Mortality		366 (11.9)	287 (15.1)	79 (6.6)	<0.001

## Discussion

In patients with SCAD, invasive treatment has been shown to improve coronary symptoms compared with optimal medical therapy alone; however, revascularization does not offer any benefits in terms of death, myocardial infarction or the need for subsequent revascularization [2–5].

Randomized controlled trials are the gold standard for evaluating therapeutic efficacy; however, there is controversy regarding the applicability of such findings to daily practice. For example, it has been reported that as many as 50 % of patients with myocardial infarction in the real world might not be represented in randomized clinical trials [24].

Both selection bias and risk burden are crucial in determining the utility of revascularization in a heterogeneous group of patients with symptomatic SCAD. We aimed to answer the question regarding how much of our population with SCAD who underwent stent implantation would have been excluded from the ISCHEMIA trial.

Based on our analysis, it should be noted that the results of the ISCHEMIA trial may not be extrapolated to a wide spectrum of patients, including subgroups of patients with moderately impaired left ventricular function, heart failure, previous myocardial infarction and revascularization, or severe angina symptoms. We want to stress the importance of registries as valuable data sources. Registries include data from all-comers, or the real-world population; therefore, an assessment of the potential benefits of different treatment modalities on a wider spectrum of patients is possible. However, there is no better way to establish proper management strategies for high-risk patients than randomized controlled trials.

Out of 938 patients excluded owing to prior revascularization within 12 months, 789 underwent percutaneous coronary intervention as part of a staged revascularization after myocardial infarction. The second part of revascularization is performed after the acute phase of myocardial infarction and symptoms are consistent with symptoms reported by patients suffering from SCAD only; percutaneous coronary intervention is performed only if the lesion is significant. Therefore, we are convinced that analysis of this subgroup is essential.

### Study limitations

It is important to note that the results of this study represent only a single-center experience. Despite the fact that a structured medical interview regarding patients’ detailed medical histories and symptoms on admission has been mandatory for an attending physicians since 2006, this was a retrospective observational study with several intrinsic limitations. Moreover, as we are a cardiology referral center in Poland, a larger proportion of high-risk patients may have been cared for at our center than in other centers. However, the application of the current guidelines and treatment methods is also higher than average in Poland.

## Conclusions

Our single-center comprehensive analysis has raised concerns regarding the fact that the ISCHEMIA trial does not represent a real-world heterogeneous group of patients with SCAD. The majority of our patients who met exclusion criteria as defined in the ISCHEMIA trial had undergone prior percutaneous coronary intervention or coronary artery bypass graft within the previous 12 months, or had a low ejection fraction, and therefore had a worse long-term prognosis. New randomized trials may be necessary, to determine the benefits of revascularization among high-risk patients with SCAD.

## Additional file

Additional file 1:
**Multivariate analysis of independent predictors of mortality in the analyzed group.** To identify predictors of long-term outcome, Cox regression models were utilized to evaluate the association between clinical laboratory electrocardiographic and angiographic variables and mortality. The stepwise selection of model building was used, with *P* = 0.1 for a confounder to stay in the model. (PDF 276 kb)

## Abbreviations

COURAGE: Clinical Outcomes Utilizing Revascularization and Aggressive Drug Evaluation
ISCHEMIA: International Study of Comparative Health Effectiveness with Medical and Invasive Approaches
SCAD: stable coronary artery disease
