# Supplementary material for: Who is eligible for randomized trials? A comparison between the exclusion criteria defined by the ISCHEMIA trial and 3102 real-world patients with stable coronary artery disease undergoing stent implantation in a single cardiology center
Source: Trials. 2015 Sep 15;16:411. doi: 10.1186/s13063-015-0934-4 (PMC4570660; doi:10.1186/s13063-015-0934-4)
Supplement: Additional file 1: — Multivariate analysis of independent predictors of mortality in the analyzed group. To identify predictors of long-term outcome, Cox regression models were utilized to evaluate the association between clinical laboratory electrocardiographic and angiographic variables and mortality. The stepwise selection of model building was used, with P = 0.1 for a confounder to stay in the model. (PDF 276 kb) [file 13063_2015_934_MOESM1_ESM.pdf]

**Independent predictors of mortality in analyzed group.**

| Factor | RR [95% CI] |
|---|---|
| Statin treatment | RR=0.98 [95%CI 0.96-1.00, p<0.001] |
| HGB (per 1mmol/l) | RR=1.03 [95%CI 0.99-1.07, p=0.076] |
| DM treated with diet | RR=2.66 [95%CI 1.15-6.13, p=0.698] |
| DM treated with insulin | RR=2.73 [95%CI 0.88-8.51, p=0.98] |
| Age (per 1year) | RR=2.73 [95%CI 0.88-8.51, p<0.001] |
| NYHA 3 or 4 class | RR=2.73 [95%CI 0.88-8.51, p=0.015] |
| DM treated with oral agents | RR=2.73 [95%CI 0.88-8.51, p=0.001] |
| PVD | RR=2.73 [95%CI 0.88-8.51, p=0.001] |
| eFGR <30 (per 1ml/min/m2) | RR=2.73 [95%CI 0.88-8.51, p=0.057] |
| ULMD | RR=2.73 [95%CI 0.88-8.51, p=0.004] |
| Severe valvular disease | RR=2.73 [95%CI 0.88-8.51, p=0.001] |
| LVEF <35% (per 1%) | RR=2.73 [95%CI 0.88-8.51, p<0.001] |
| Disease limiting survival | RR=2.73 [95%CI 0.88-8.51, p<0.001] |

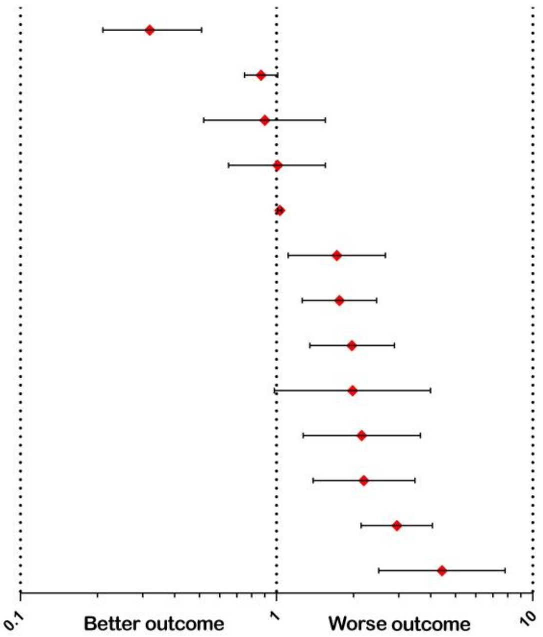


HGB – hemoglobin; DM – diabetes mellitus; NYHA – New York Heart Association class; PVD – peripheral vessel disease; eGFR – estimated Glomerular Filtration Rate; ULMD – unprotected Left Main disease; LVEF – left ventricle ejection fraction.
